# Use of national-scale data to examine human-mediated additions of heavy metals to wetland soils of the US

**DOI:** 10.1007/s10661-019-7315-5

**Published:** 2019-06-20

**Authors:** Amanda M. Nahlik, Karen A. Blocksom, Alan T. Herlihy, Mary E. Kentula, Teresa K. Magee, Steven G. Paulsen

**Affiliations:** 1 Office of Research and Development, National Health and Environmental Effects Research Laboratory, Western Ecology Division, United States Environmental Protection Agency, Corvallis, OR USA; 2 Department of Fisheries and Wildlife, Oregon State University, 104 Nash Hall, Corvallis, OR 97331 USA

**Keywords:** National Wetland Condition Assessment (NWCA), Trace elements, Lead (Pb), Background concentrations, Heavy Metal Index (HMI), Anthropogenic disturbance

## Abstract

Soil concentrations of 12 heavy metals that have been linked to various anthropogenic activities were measured in samples collected from the uppermost horizon in approximately 1000 wetlands across the conterminous US as part of the 2011 National Wetland Condition Assessment (NWCA). The heavy metals were silver (Ag), cadmium (Cd), cobalt (Co), chromium (Cr), copper (Cu), nickel (Ni), lead (Pb), antimony (Sb), tin (Sn), vanadium (V), tungsten (W), and zinc (Zn). Using thresholds to distinguish natural background concentrations from human-mediated additions, we evaluated wetland soil heavy metal concentrations in the conterminous US and four regions using a Heavy Metal Index (HMI) that reflects human-mediated heavy metal loads based on the number of elements above expected background concentration. We also examined the individual elements to detect concentrations of heavy metals above expected background that frequently occur in wetland soils. Our data show that wetland soils of the conterminous US typically have low heavy metal loads, and that most of the measured elements occur nationally in concentrations below thresholds that relate to anthropogenic activities. However, we found that soil lead is more common in wetland soils than other measured elements, occurring nationally in 11.3% of the wetland area in concentrations above expected natural background (> 35 ppm). Our data show positive relationships between soil lead concentration and four individual landscape metrics: road density, percent impervious surface, housing unit density, and population density in a 1-km radius buffer area surrounding a site. These relationships, while evident on a national level, are strongest in the eastern US, where the highest road densities and greatest population densities occur. Because lead can be strongly bound to wetland soils in particular, maintenance of the good condition of our nation’s wetlands is likely to minimize risk of lead mobilization.

**Electronic supplementary material:**

The online version of this article (10.1007/s10661-019-7315-5) contains supplementary material, which is available to authorized users.

## Introduction

The use of heavy metals is ingrained in human culture. Lead, for example, was one of the first metals to be used by man, and archeological discoveries date the earliest cast lead objects and lead pigments to approximately 7000 BCE (Lessler 1988). Copper has a history of use that extends past the Roman era and back to 5000 BCE, when the first known instances of mining and smelting occurred (Oorts 2013). Chromium use dates back to more than 2000 years ago, as evidenced by archeological finds of Chinese chrome-plated bronze weapons (Gonnelli and Renella 2013). Scientific and technological progress in the last several centuries have resulted in the discovery and use of modern metals, such as tungsten and cadmium (Krebs 2006). Beginning in the twentieth century and continuing to today, advances in metal mining and smelting operations and more efficient production yields of metals are resulting in more widespread use of heavy metals (Han et al. 2002; Callender 2003). It is estimated that more than 95% of all copper ever extracted has been mined and smelted since 1900 (Oorts 2013), zinc and nickel production more than doubled between 1973 and 2010 (Alloway 2013), chromium production has increased exponentially from 1970 to 2002 (Han et al. 2002), and the annual global production of tungsten has increased from virtually zero in 1905 to over 70,000 tonnes in 2013 (Dvořáček et al. 2017)—evidence of the increasing extent to which we rely on heavy metals in our modern lives.

The consequence of extensive and seemingly ceaseless use of heavy metals by humans is dispersal of heavy metals, ultimately, into our soils (Han et al. 2002; Callender 2003). The linkage between modern anthropogenic activities and release of heavy metals into our environment is well documented. Background levels of trace elements in soils have been reported in the US (e.g., Shacklette and Boerngen 1984; Holmgren et al. 1993) and in other countries (e.g., Andersen et al. 1994; McGrath and Zhao 2006; Alfaro et al. 2015; Shifaw 2018), with elevated levels of heavy metals in soils linked to various anthropogenic uses and activities ranging from industry to roads to agriculture (Nriagu and Pacyna 1988; Alloway 2013). Furthermore, soils represent the most concentrated physical pool of metals in aquatic environments (Luoma 1983), yet concentrations of soil heavy metals are not well documented over large spatial scales for aquatic environments—especially wetlands, which are often located in optimal locations to intercept surface water and sediments. Being able to distinguish naturally occurring soil heavy metal concentrations from human-mediated heavy metal additions to our wetland ecosystems at national and regional scales, even in concentrations below biotic toxic effects, is important for (a) determining patterns of soil heavy metal concentrations on the aquatic landscape, (b) identifying heavy metals in aquatic soils that occur frequently in concentrations above expected background, and (c) anticipating the need for future management actions.

In 2011, the United States Environmental Protection Agency (US EPA) conducted the first National Wetland Condition Assessment (NWCA), a national survey of wetlands across the conterminous US based on a probability design (Kentula and Paulsen 2019). The results of other survey designs, such as those that use sampling locations across large areas that are evenly distributed, randomly selected, hand-selected, or based on convenience/access, are typically expressed as arithmetic means, geometric means, or medians and ranges that are aggregations of individual sites sampled. Alternatively, a probability design uses sampling locations that provide a sample of a well-defined population through the use of weights (i.e., the number of acres of the population represented by each site) to generate results expressed as estimates of the entire population (Olsen and Peck 2008; Olsen et al. 2019).

As part of the 2011 NWCA, soil concentrations of 12 heavy metals were measured in approximately 900 probability-selected wetlands across the conterminous US. These data represent the first wetland soil heavy metal concentrations collected using a survey designed to report across the wetland population of the US at both national and regional scales. We developed national thresholds for each measured element to distinguish naturally occurring soil heavy metal concentrations from human-mediated heavy metal additions to our wetland ecosystems. While examining soil heavy metal concentrations by individual element is important for understanding detailed patterns and identifying heavy metals in aquatic soils that occur frequently in concentrations above expected background, aggregating the 12 heavy metals measured into an index can be a useful way to identify the magnitude of human activities that could negatively affect the wetland population across the nation and regions. A single-number index can also be a simple and effective way to communicate results to the public and policy makers. Ultimately, both analyses aid in anticipating the need for future management actions through risk analyses, like those employed by Herlihy et al. (2019a).

**Fig. 1:**
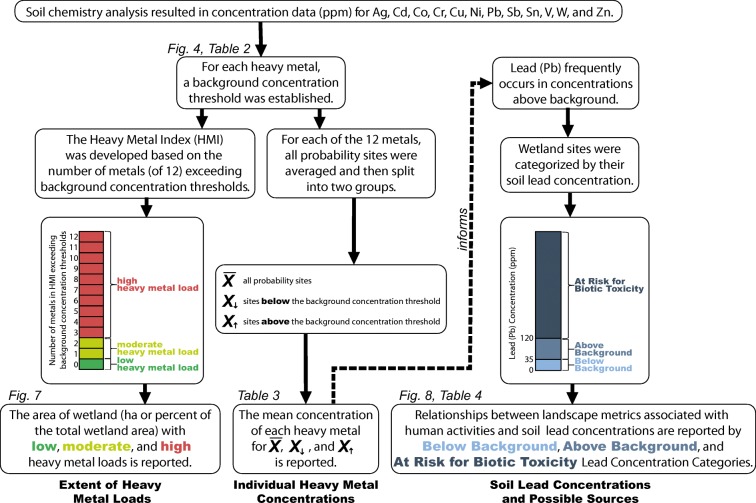
An organizational flow chart summarizing the data, analysis, and results that are reported in this article. Titles in bold at the bottom of the flow chart refer to the “Results and discussion” subsection in which the results of the analyses are presented and discussed. Note that all results are probability-weighted

In this article, we distinguish natural background concentrations from human-mediated additions to evaluate wetland soil heavy metal concentrations in the conterminous US and four regions in two different ways. First, we combine the 12 elements into a Heavy Metal Index (HMI) that reflects human-mediated heavy metal loads based on the number of elements above expected background concentration, and then we examine individual elements to detect concentrations of heavy metals above expected background that frequently occur in wetland soils (Fig. 1). Finally, based on our national results of individual heavy metal concentrations in wetlands, we provide an in-depth examination of soil lead concentrations and possible sources, as lead was the most frequently occurring heavy metal nationally in the NWCA.

## Methods

### Survey design

**Fig. 2:**
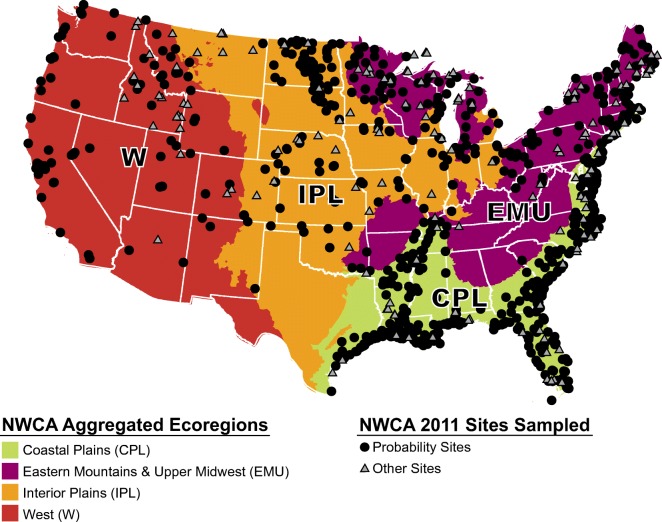
The distribution of probability sites and other sites (i.e., sites that met the target definition but were selected using a method other than the national probability design) sampled as part of the 2011 National Wetland Condition Assessment (NWCA)

In 2011, 1138 wetland sites within four NWCA Aggregated Ecoregions (also referred to as “regions”) in the contiguous US were sampled as part of the NWCA, an effort to report on the condition of our nation’s wetlands led by US EPA in cooperation with states and tribes (US EPA 2016a; Fig. 2). Of these, 967 sites that met the definition for the target population—“all wetlands of the conterminous US not currently in crop production, including tidal and non-tidal wetted areas with rooted vegetation and, when present, shallow, open water less than 1 m in depth” (US EPA 2011a)—were selected from the US Fish and Wildlife Service’s National Wetland Status and Trends sample frame (Dahl and Bergeson 2009; Dahl 2011) using a probability design based on methods described in Olsen and Peck (2008) and Olsen et al. (2019). To ensure that each state had sites to sample and that all wetland types were represented, coordinates (i.e., latitude and longitude; hereafter referred to as the “point”) of the individual sites to be sampled were selected using state as a stratum with unequal probability of selection by seven wetland types, as discussed in Olsen et al. (2019). These 967 sites are hereon referred to as “probability sites.” In addition to the 967 probability sites, an additional 171 sites (hereon referred to as “other” sites) that met the target definition but coordinates of which were selected using a method other than the national probability design were sampled (Herlihy et al. 2019b). Approximately 10% of the probability sites (96 sites)—at least two from each state—were selected to be revisited (i.e., resampled) to gauge temporal variability within the sampling period and for quality assurance (QA) (Kaufmann et al. 1999; Stoddard et al. 2008; Table 1). These 96 sites are hereon referred to as “revisit” sites.

**Table 1:** The *n* values associated with the representative soil pits for probability sites and other sites (i.e., sites that met the target definition but were selected using a method other than the national probability design)

	Probability	Other	Total
Sampled sites	967	171	1138
Revisited sites	96	0	96
Sampled sites with soil profile data	916	165	1081
Sampled sites missing soil profile data	51	6	57
Soil horizons described as part of soil profile data	3480	581	4061
Sampled sites with soil chemistry data	874	165	1039
Sampled sites missing all soil chemistry data	93	6	99
Sampled sites missing the top horizon of soil chemistry data	238	35	273
Soil horizons analyzed as part of soil chemistry data	2699	481	3180

### Field sampling

Field crews conducted 1-day visits to each of the 1138 wetland points during the 2011 growing season (April through September, depending on location) (US EPA 2011a). Upon arrival at a point, a 0.5-ha circular assessment area (AA) was established by measuring a 40-m radius from the point, and adjusting the location to assure that no more than 10% of the area of the AA was in upland or in water over 1 m deep. In cases where a point fell into a narrow wetland or a wetland smaller than 0.5 ha, the shape or area of a standard, circular 0.5-ha AA was adjusted (to no less than 0.1 ha).

**Fig. 3:**
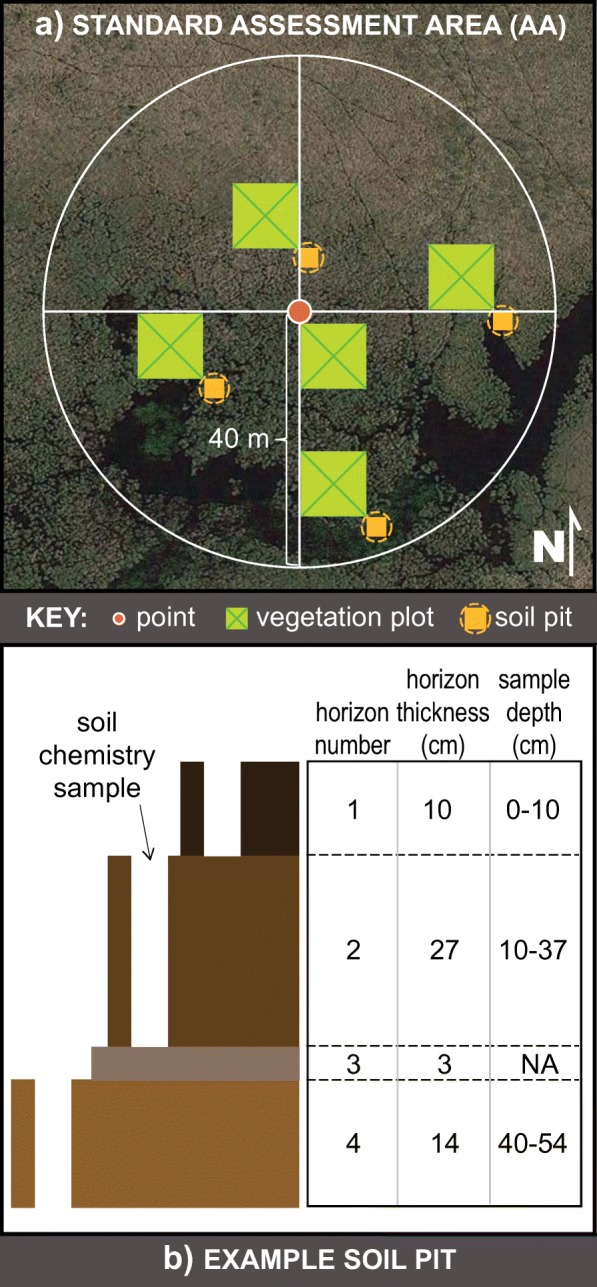
**a** Location of vegetation plots and soil pits within a standard assessment area (AA) and **b** an example of the upper horizons of a representative soil pit, designating how soil chemistry samples were to be collected within the horizons. Note that Horizon 3 in the example soil pit would not be sampled for soil chemistry because it is less than 8 cm thick

Using protocols outlined in the 2011 NWCA Field Operations Manual (US EPA 2011a), four soil pits were established within the AA of each site at the southeast corners of the four vegetation plots furthest from the point (Fig. 3a). Because of the wide range of soil types and characteristics encountered across sites, field crews were instructed to use their best professional judgment to choose the appropriate tools to excavate. Each of the soil pits were excavated to a depth of 60 cm, typically using a tiling, sharpshooter, or pointed-tip shovel. During this excavation, field crews recorded descriptive soil profile data, including depth and thickness, soil texture, soil matrix color, and redoximorphic/organic/mottle features, for each horizon (US EPA 2011a). One soil pit was randomly selected from the subset of pits most representative of the soil conditions of the AA, then excavated to a maximum depth of 125 cm, typically using a shovel, a bucket auger, or a tube extractor (for unconsolidated substrate). Field crews continued to record descriptive data for the deeper horizons. In some cases, site characteristics (e.g., unconsolidated soil, inundation, shallow bedrock) physically prohibited field crews from excavating the soil pit to a depth of 125 cm, in which case the representative pit was dug as deep as possible. For every horizon equal to or greater than 8-cm thick at the representative pit (surface to a depth of 125 cm or as deep as possible), a soil sample (comprised of 1 to 2.5 L of soil) from horizon boundary-to-boundary was collected using a shovel or auger (Fig. 3b). In cases where the surface horizon was less than 8 cm thick, field crews were instructed to collect a combined soil sample of the top two horizons.

Ultimately, field crews were able to collect soil samples from 874 probability sites and 165 other sites. Ninety-nine sites (93 probability sites and 6 other sites) were not sampled due to site constraints (e.g., deep water, unconsolidated soils, and shallow bedrock) (Table 1). The number of soil horizons at each site ranged from 1 to 9, with horizon thicknesses ranging from 1 to 170 cm. Approximately one fifth of the described soil horizons (881 of 4061 horizons) from the probability and other sites were not sampled for soil chemistry, many of which were less than 8 cm thick and, therefore, excluded for soil chemistry as instructed in the NWCA Soils Protocol (US EPA 2011a).

Field crews kept soil samples as cool as possible and out of direct sun while in the field. Soils were stored and shipped in batches to the Natural Resources Conservation Service (NRCS) Kellogg Soil Survey Laboratory (SSL) in Lincoln, Nebraska, for analysis.

### Soil sample analyses

#### Trace elements

Soil samples were analyzed for trace elements using standard NRCS-SSL procedure 4H1a1a1a1-20, which follows US EPA Method 3051A, and consists of a microwave acid digestion followed by inductively coupled plasma–atomic emission spectrophotometry (ICP-AES) (US EPA 2011b; Soil Survey Staff 2004). This approach maximizes the extractable concentration of elements in digested soils while minimizing matrix interferences that can occur in digestion procedures using hydrofluoric acid. The concentration of silver (Ag), cadmium (Cd), cobalt (Co), chromium (Cr), copper (Cu), nickel (Ni), lead (Pb), antimony (Sb), tin (Sn), vanadium (V), tungsten (W), and zinc (Zn) were determined using an ICP-AES. Results were reported to the nearest 0.01 mg kg^−1^ (note that mg kg^−1^ is equivalent to ppm).

#### Soil pH

Soil pH was measured in a soil-water (1:1) solution using standard NRCS-SSL procedure 4C1a2a1a-b1 (US EPA 2011b; Soil Survey Staff 2004). An air-dried, finely-ground, 20-g soil sample was mixed with 20 mL of reverse-osmosis water. After an equilibration period of 1 h with occasional stirring, the sample was stirred for 30 s and the water pH was measured to the nearest 0.1 pH unit. Individual pH values were back-transformed to [H^+^] before calculating national and regional means.

#### Soil organic carbon

To prepare samples for carbon analysis, soils were air dried, crushed, and sieved to < 2 mm to obtain the fine earth fraction. Total carbon was measured using an elemental analyzer (standard NRCS-SSL procedure 4H2a1-3), and inorganic carbon (i.e., calcium carbonate (CaCO_3_) equivalent) was determined by exposing the soils to hydrochloric acid (HCl) and measuring the evolved carbon dioxide (CO_2_) manometrically using standard NRCS-SSL procedure 4E1a1a1a1-2 (US EPA 2011b; Soil Survey Staff 2004). Soil organic carbon was calculated as the difference between total and inorganic carbon and is reported in percent (Nahlik and Fennessy 2016).

### Data QA

Trace element data returned from NRCS were merged with soil profile data collected by field crews from the representative pit (i.e., the only pit from which soil was analyzed for chemistry). The soil chemistry database, consisting of soil horizons from the representative pits and associated soil chemistry for sites sampled, was thoroughly inspected using a QA process for internal consistency and data entry errors.

NRCS performed internal QA on soil chemistry data, flagging any data below the practical quantitation limit (PQL) or minimum detection limit (MDL) of the equipment used to analyze the samples (US EPA 2011b). Aside from identifying which samples were below limits, the flags also specified the limits for each analyte. Values below the MDL were changed to half the specified MDL in this dataset. All detection limits were below our established background concentration thresholds (see the following section for details).

Signal-to-noise ratio (S:N) is a measurement of repeatability that compares the ratio of the variance among all sites (the signal) to the variance within site from the revisits (the noise) calculated by random effect analysis of variance (Kaufmann et al. 1999). S:N for each element was calculated using a model that included all 1138 sites (the signal) and the 96 revisit sites sampled twice during the field season (the noise) (Table 1). Noise includes temporal, sampling, and laboratory variabilities. Elements with S:N ≤ 1 indicate that the measurement was associated with as much or more variability as sampling two different sites (Stoddard et al. 2008), and Kaufmann et al. (2014) report that the adverse effects of noise variance on data analyses are negligible when S:N > 10 and minor as S:N decreases to 6.

### Development of the HMI

Based on the NWCA soils protocol (US EPA 2011a), field crews were instructed to collect soil samples from boundary to boundary of each horizon (Fig. 3b). Despite some difficulties in consistently sampling the first horizon (i.e., Horizon 1 was less than 8 cm thick at about one quarter of the probability and other sites (273 of 1039 sites) and erroneously not sampled (Table 1)), examination of the data showed that every site with soils data had at least one horizon with soil chemistry measured within 50 cm of the surface. Because the upper part of the soil is the most biologically active (US EPA 2015a) and most indicative of human impacts in and around the AA, concentrations of 12 elements measured in the uppermost horizon, typically within 10 cm of the soil surface, were used to develop the HMI. The heavy metals included in the HMI, their primary anthropogenic associations, and examples of specific anthropogenic sources are reported in Table 2.

**Table 2:** Summary of the characteristics of the trace elements used in the Heavy Metal Index (HMI), primarily from information reported in Alloway (2013)

Element	Primary anthropogenic associations	Examples of specific anthropogenic sources	Published natural background concentration (ppm)	Background concentration threshold (ppm)
Silver (Ag)	Industry	• Various industrial operations • Nanoparticle contamination	0.05–1.00	1.0
Cadmium (Cd)	Agriculture	• Phosphate fertilizers • Iron and steel production • Oil combustion	0.1–1.0	1.0
Cobalt (Co)	Industry	• Industrial application of oxides, hydrous oxides, or arsenides	< 50	25
Chromium (Cr)	Industry/agriculture	• Stainless steel production • Sewage sludge • Fly ash • Slag	0.5–250	125
Copper (Cu)	Agriculture/industry/roads	• Manure • Sewage sludge • Phosphate fertilizers • Agricultural pesticides • Atmospheric deposition from volcanic eruptions, forest fires, sea-salt spray • Metal production • Fossil fuel combustion • Brake and tire wear from cars and railroads	2–50	50
Nickel (Ni)	Industry/agriculture	• Metal-processing emissions • Coal/oil combustion • Sewage sludge • Phosphate fertilizers	0.2–450	225
Lead (Pb)	Roads/industry	• Leaded gasoline • Smelting of base ores • Ammunition • Sewage sludge	Mean of 17^a^	35
Antimony (Sb)	Industry	• Smelting of base ores • Flame retardant and catalyst in plastics • Ammunition	0.1–1.9	1.0
Tin (Sn)	Industry/agriculture	• Marine antifouling paints • Agricultural pesticides • Industrial fungicides • Slimicides • Wood preservatives	1.7–50	17
Vanadium (V)	Industry/roads	• Coal/oil combustion • Petroleum products	36–150	150
Tungsten (W)	Industry/agriculture	• Mining • Various industrial operations • Military operations • Ammunition • Household waste disposal (e.g., lightbulbs)	< 2	2.0
Zinc (Zn)	Industry/agriculture	• Fossil fuel combustion • Atmospheric deposition from volcanic eruptions, forest fires • Inorganic fertilizers • Manure • Sewage sludge	10–150	150

**Fig. 4:**
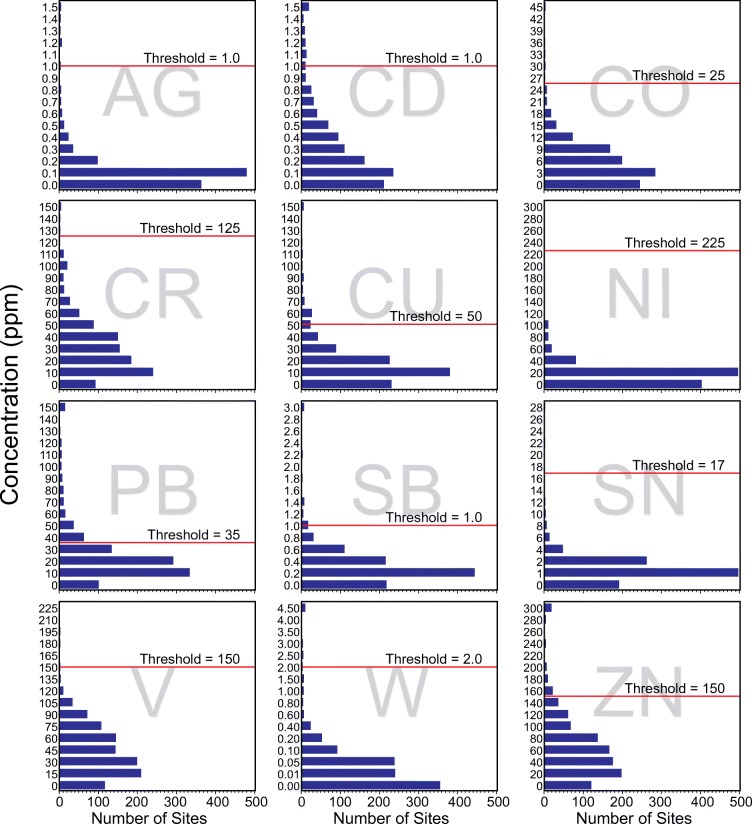
Frequency histograms of the concentrations of each measured element in this study for probability and other sites with soil chemistry data (*n* = 1039), used to set expected background concentration thresholds (designated by the red lines) in combination with published background concentration ranges from Alloway (2013). The full names of the elements are reported in the “Methods” section and in Table 2

Background concentration thresholds are based on the published natural background concentrations and adjusted for consistency with natural breaks in our data (see Fig. 4). Thresholds are used to reflect human-mediated additions of heavy metals to wetland soils

^a^Alloway (2013) publishes a global mean for Pb, reported in this table, which is consistent with means determined for the conterminous US by Shacklette and Boerngen (1984)

The HMI, originally developed and peer-reviewed as part of the 2011 NWCA (US EPA 2016a, b), was created using soil chemistry data from both probability and other sites (i.e., 1039 sites) (Table 1). The HMI was scored as the sum of the number of heavy metals present at a site with concentrations above a set threshold, with higher HMI values indicating greater human influence at a site. Given that there are 12 elements included in the HMI, the range of possible scores is 0 to 12. We used an equal weighting of 1 assigned to each element because potential effects of human-mediated inputs of heavy metals to wetland soils are often additive (Swartz et al. 1988; Fairey et al. 2001; Chu and Chow 2002; Norwood et al. 2003). To be clear, these thresholds were designed to be assessment tools and *do not* indicate human health responses, biological responses, mobilization of heavy metals, or ecological condition—these are research questions that are outside the scope of this dataset and this study. However, the additive approach we use for the HMI is similar to that used to develop sediment quality guidelines (SQGs), which are used in monitoring and assessment to predict when chemical concentrations are likely to be associated with a measurable biological response (e.g., Long et al. 1998; Fairey et al. 2001).

When setting thresholds, we aimed to establish a single, national threshold for each element, in part because NWCA field protocol is designed to report primarily on national and regional scales. We chose to use national instead of regional thresholds because comparing results across regions using differing regional thresholds becomes problematic. Secondly, given the national scale at which we sample, the variability among sites is likely to be much greater than the degree of error in assigning national thresholds. So, even though some precision is sacrificed at a site scale, the thresholds we developed are likely to be generally correct when reporting on national and regional scales. Finally, when creating thresholds, it is imperative to have a sample size large enough to be representative of the reporting scale. While we recognize that background concentrations of heavy metals naturally vary depending on soil type, underlying geology, soil chemistry, etc., setting more site-specific thresholds is beyond the scope of the NWCA and this study. Also, our data did not support a separate study to establish new background concentrations of elements in wetland soils.

We established a single, national threshold for each element to indicate human-mediated additions of heavy metals to wetland soils based on natural background concentrations (i.e., soils with minimal anthropogenic inputs). Specifically, we relied on a combination of the published ranges of natural background concentrations of elements in terrestrial soils (or saturated soils, if available) reported in Alloway (2013) and the distribution of heavy metal concentrations at our sites to establish thresholds (Table 2). Within the published ranges reported in Alloway (2013), we examined histograms of heavy metal concentrations and set thresholds based on breaks in our data (Fig. 4). This resulted in establishment of background concentration thresholds that were within reported ranges by Alloway (2013) (Table 2), albeit above most of the reported means for terrestrial soils reported by Shacklette and Boerngen (1984). However, given that background concentrations of elements in wetland soils have never been measured on a national scale and that many heavy metals have an affinity for organic matter (Förstner and Wittmann 1981; Lin and Chen 1998), which would likely make background concentrations higher in many wetland soils than in terrestrial soils, we used the middle to high end of reported ranges in Alloway (2013) to guide the establishment of wetland background concentration thresholds.

### Heavy metal load categories based on HMI

**Fig. 5:**
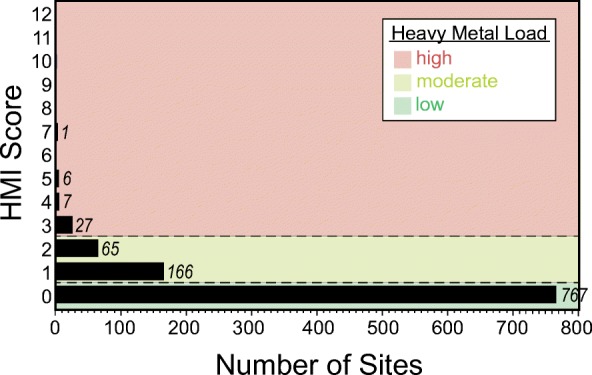
Frequency histogram of Heavy Metal Index (HMI) scores for probability and other sites with soil chemistry data (*n* = 1039), used to set the thresholds (designated by the horizontal lines) for low, moderate, and high heavy metal load categories. Numbers following each bar show the number of sites with the HMI Score

The HMI scores are used to report the areal extent of wetlands with low, moderate, and high heavy metal loads. The heavy metal load categories are a communication tool; thus, low, moderate, and high heavy metal loads were defined relative to the range of scores found in our sampled sites (Fig. 5). We equated low heavy metal loads with an HMI score of 0, meaning that all 12 element concentrations were equal to or below the background concentration threshold established for each element. Moderate heavy metal loads included HMI scores of 1 and 2, and, finally, high heavy metal loads were represented by HMI scores of 3 or above (i.e., three or more element concentrations were above the background concentration thresholds).

### Individual heavy metal concentrations

Using the same background concentration thresholds established for the HMI (Table 2), the national and regional population-weighted mean soil concentrations of each individual element included in this study were calculated for all sites ($\overline{X}$), as well as for the sites below the background concentration threshold (*X*_↓_), and sites above the background concentration threshold (*X*_↑_). In this study, elements that exceeded concentrations above the established threshold (*X*_↑_) in more than 5% of the national or regional wetland area were considered common, or frequently occurring.

### Soil lead concentrations and possible sources

Relationships among lead concentrations in the uppermost horizon with soil chemistry data and landscape metrics associated with human activities were investigated nationally and for the four NWCA Aggregated Ecoregions. Each probability site was assigned to a lead concentration category, with “Below Background” indicating a soil concentration ≤ 35 ppm Pb, “Above Background” indicating a soil concentration 36–119 ppm Pb, and “At Risk for Biotic Toxicity” indicating a soil concentration ≥ 120 ppm Pb. The concentration ≤ 35 ppm Pb for the Below Background category is consistent with the background concentration threshold used for Pb in the HMI and natural background concentrations (Alloway 2013), while the At Risk for Biotic Toxicity category threshold was set based on US EPA ecological soil screening levels (Eco-SSLs) of ≥ 120 ppm Pb, which represents the geometric mean of the maximum acceptable toxicant concentration (MATC) value for four test species of terrestrial plants (aquatic plants were not included in the study) under three different soil pH and percent organic matter conditions (US EPA 2005).

Landscape metrics were calculated using ArcGIS software developed by Esri (2014 release) and available GIS data layers for the area within a 1-km-radius buffer (hereon referred to as the “1-km buffer”) surrounding the selected wetland point, and included four metrics: road density, impervious surface cover, population density, and housing unit density. Road density, reported in km km^−2^ for the 1-km buffer, was developed using 2010 TIGER (US Census Bureau 2010) and NPScape (National Park Service 2014) data. The road density was calculated for 1-km raster cells and was resampled to 100 m for the metric computations. Impervious surface cover was developed using the 2006 National Land Cover Data (NLCD; Fry et al. 2011) and represents the percentage of impervious surface for each 30-m raster cell in the NLCD impervious surface raster, and raster cells of percent impervious were summarized for the 1-km buffer. Population density and housing unit density were developed using Census 2010 Block Group population data (US Census Bureau 2010), represented in 90-m raster grid cells and summarized for the 1-km buffer, and reported as people mi^−2^ and housing units mi^−2^, respectively. For all four landscape metrics, the means for each geometry were computed using a tool that automatically handles the overlapping buffer geometries and uses ArcGIS zonal statistics to compute the necessary statistics. Population-weighted means for each of the landscape metrics were calculated for the nation by lead concentration category.

### Statistics and data visualization

The probabilistic design assigns sample weights to each of the 967 individual probability sites based on the inverse probability of the point being sampled (Stevens Jr. and Olsen 1999, 2000, 2004; Olsen et al., 2019) so results may be expressed as estimates of the entire resource by area of sampled wetlands (i.e., extent), which was 25.2 million ha. All results (i.e., extent of heavy metal loads, baseline heavy metal concentration means, and lead concentration category means) are weighted and, thus, are inferable to the national NWCA target population. Extent estimates are also reported for four NWCA Aggregated Ecoregions (Coastal Plains, Eastern Mountains & Upper Midwest, Interior Plains, and West) (Fig. 2).

Ninety-three probability sites were missing soil chemistry data primarily due to site conditions (e.g., presence of deep surface water or other site conditions on the sampling day) or laboratory issues (e.g., 45 soil samples from sites in Interior Plains were not analyzed using NWCA laboratory protocols) and were therefore excluded from heavy metal load assignments. The extent of wetland area represented by these incompletely sampled sites is reported as missing in the national and regional results.

The population-weighted statistical estimates of the extent of heavy metal loads, baseline heavy metal concentration means, and lead concentration category means for the national population and regional subpopulations of wetlands were completed with data for Visit 1 of the probability sites using the *spsurvey* package in R version 3.3.0 (Kincaid and Olsen 2015; R Core Team 2016). All statistical calculations use the sample weight to calculate the means and known margins of error (two-sided 95% confidence interval (CI)) based on a local neighborhood variance estimate (Stevens Jr. and Olsen 2004). The 95% CI was used to identify significant differences among population-weighted means. In addition to population-weighted means, areal extent (in hectares and as a percent of the wetland population) is reported in the tables and figures. Graphics were constructed in R using the *ggplot2* package (Wickham 2009) and imported to Adobe Illustrator CC (version 19.2.1) for final editing.

## Results and discussion

**Fig. 6:**
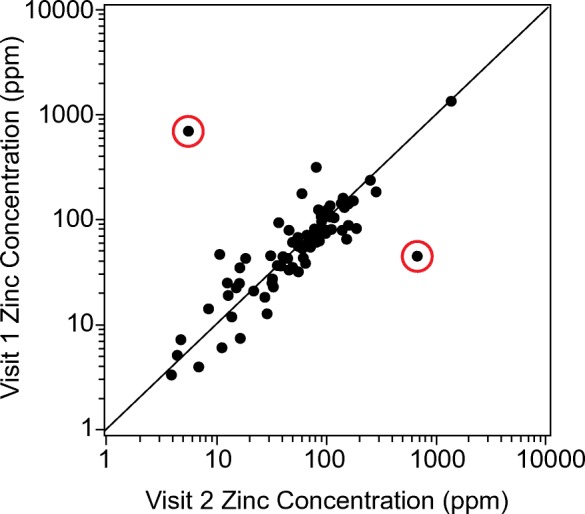
Zinc concentrations (ppm) measured between Visit 1 and Visit 2. The sites circled in red represent outliers that are likely affecting the signal-to-noise ratio (S:N) for this element

Soil trace element concentrations for 12 heavy metals—silver (Ag), cadmium (Cd), cobalt (Co), chromium (Cr), copper (Cu), nickel (Ni), lead (Pb), antimony (Sb), tin (Sn), vanadium (V), tungsten (W), and zinc (Zn)—were analyzed in the uppermost horizon at 874 probability sites across the conterminous US. The uppermost horizon represented a mean thickness of 29.18 ± 1.69 cm with the top depth typically beginning within the top 2 cm of the soil surface (mean of 1.80 ± 0.27 cm) at the sites. Specifically, 125 of the 874 probability samples represented a horizon that began and ended within 10 cm of the soil surface, and 850 of the 874 probability samples represented a horizon that began in the top 10 cm of the surface (and on average ended at 30.93 cm from the soil surface). The S:N for the measured elements ranged from 1.38 (Zn) to 230.62 (W) (Table 3), indicating that adverse effects of noise variance were negligible or minor for most elements. The S:N for Zn was particularly low, suggesting that there may be adverse effects of noise variance on data analyses. Further investigation of Zn revealed that two paired samples (from Visit 1 and Visit 2 at the same site (Fig. 6)) out of the 96 revisits were driving the low S:N, likely due to sample contamination in the field (e.g., sunscreen).

**Table 3:**
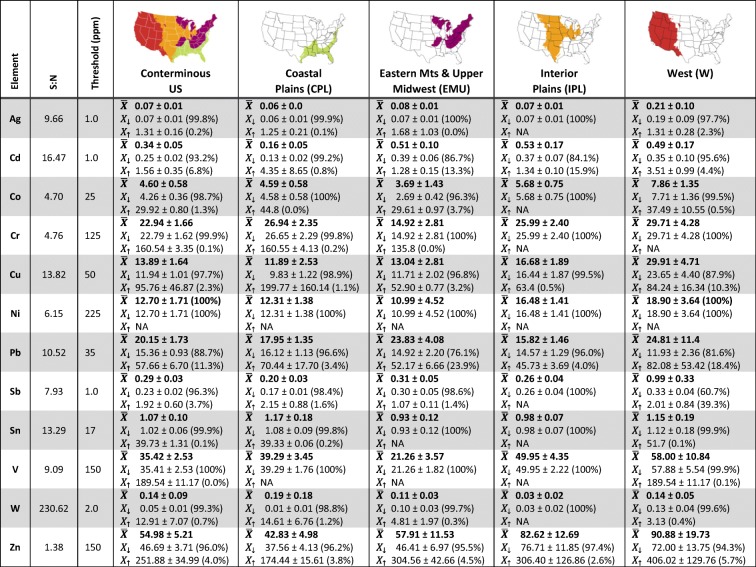
For each of the 12 measured elements, the signal-to-noise ratio (S:N), the background concentration threshold (ppm), and mean concentration (ppm) ± 95% confidence interval (CI) in the uppermost horizon with soil chemistry. Concentrations are expressed as population means for all probability sites $\left(\overline{X}\right)$and for probability sites with heavy metal concentrations below (*X*_↓_) and above (*X*_↑_) the background concentration threshold. Percent of the population is in parentheses for *X*_↓_ and *X*_↑_, with $\overline{X}$representing the 100% of the sampled population. NA signifies that there were no sites with heavy metal concentrations above the threshold. Areal extent (ha) and number of sites associated with each mean are reported in Supplementary Table 1

### Extent of heavy metal loads

**Fig. 7:**
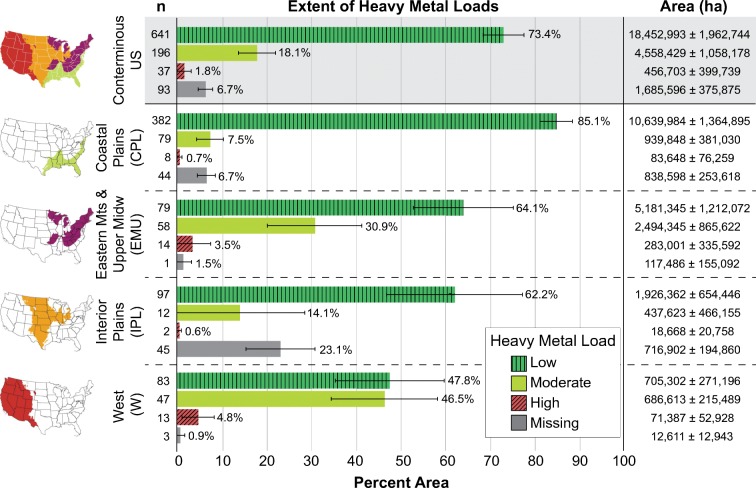
Extent of heavy metal loads reported for wetlands of the conterminous US and each of the four NWCA Aggregated Ecoregions. Heavy metal load categories include low (green vertically striped bars representing an HMI score of 0 metals above background concentration thresholds), moderate (yellow solid bars, representing an HMI score of one to two metals above background concentration thresholds), high (red diagonally striped bars, representing an HMI score of three or more metals above background concentration thresholds), and missing (gray solid bars). Percent area (represented by the bar length and numerically) is reported with the associated wetland area (ha) and the number of probability sites (*n*) included in the calculations. Error bars represent 95% CI

Twelve elements were aggregated into an HMI that reflects human-mediated heavy metal loads based on the number of elements above thresholds representing expected background concentration. The HMI was calculated for each site, and then the proportion of total area in each heavy metal load category (high, moderate, and low) was calculated, with the total wetland area representing an estimated 23.48 ± 1.90 million ha across the conterminous US. Figure 7 reports the extent of heavy metal loads for the NWCA sampled population across the conterminous US and for the four NWCA Aggregated Ecoregions.

Most of the wetland area for this sampled population had heavy metal concentrations below the established background concentration thresholds. Consequently, the majority of assessed wetland area in the nation, 73.4%, had low heavy metal loads. Moderate heavy metal loads, indicating that one or two elements included in the HMI were above the background concentration threshold, were found in 18.1% of the wetland area nationally, and high heavy metal loads (indicating that three or more elements included in the HMI were above the background concentration threshold) were found in only 1.8% of the area. Of the four NWCA Aggregated Ecoregions, Eastern Mountains & Upper Midwest and West had the greatest percentage of wetland area both with moderate heavy metal loads (30.9 and 46.5%, respectively) and with high heavy metal loads (3.5 and 4.8%, respectively). In comparison, Coastal Plains and Interior Plains only had 7.5 and 14.1% wetland area (for Coastal Plains and Interior Plains, respectively) with moderate heavy metal loads and 0.7 and 0.6% wetland area (for Coastal Plains and Interior Plains, respectively) with high heavy metal loads.

Soil samples could not be collected or were not analyzed for heavy metal concentrations for 6.7% of the area nationally (represented by gray bars in Fig. 7), with the greatest amount of missing data from Interior Plains (23.1% of wetland area), due to use of soil analysis protocols that were incongruent with those used by the NWCA, and from Coastal Plains (6.7% of wetland area), likely due to standing water that impeded soil sample collection.

To our knowledge, the HMI is the first large-scale, chemical indicator of human-mediated additions of heavy metals to wetlands. Aggregation of the 12 measured elements into the HMI supports the estimation of the magnitude of human activities (i.e., human disturbances to the wetland sites) that could negatively affect the wetland population across the nation and regions. As such, the HMI was used as a core indicator for the 2011 NWCA—first, as one criterion in identifying least-disturbed wetland sites for use as reference sites (Herlihy et al. 2019b), and then to determine the relationship between the degree of presence of heavy metals in soils and wetland condition using risk analyses (Herlihy et al. 2019a). Primary research to connect heavy metal concentrations to ecological condition is beyond the scope of the NWCA and this study; however, employing a relative risk analysis, which predicts the likelihood of wetland being in poor ecological condition when a site has high compared to low heavy metal loads as indicated by the HMI (Herlihy et al. 2019a), is an important tool for evaluating the effects of heavy metal loads on wetland condition over large scales and for anticipating the need for future management actions in particular regions or across the nation.

The HMI has limitations of which users need to be aware. For one, the methodology that we used to develop expected background thresholds for the HMI was based on published background concentration ranges for terrestrial soils and adapted to our data. Secondly, we established a single, national threshold for each measured element, even though background concentrations of heavy metals can be highly influenced by the parent material. Given that there are no established background concentrations of elements for wetland soils and that our sample size of least-disturbed (i.e., reference) wetlands was not large enough to support the establishment of wetland soil background concentrations, we set our thresholds based on the best available information (i.e., terrestrial background concentrations) to err on the side of underestimating the extent of wetlands with human-mediated heavy metal additions. We believe that at large scales (such as national or regional scales), the natural variability across sites negates the limitations of the index; however, at small scales (such as local or site scales), these limitations may reduce the utility of the HMI. These limitations also apply to the thresholds used to investigate individual heavy metal concentrations (see the following subsection). We hope to refine the thresholds as more data are gathered through future NWCA surveys. The NWCA is conducted every five years, and, over time, we plan to use the heavy metal concentrations measured in least disturbed sites (e.g., Herlihy and Sifneos 2008; Herlihy et al. 2013) to revise our thresholds. Depending on the number of least disturbed sites sampled, we may be able to revise the thresholds based on region, parent material, or other wetland characteristics.

### Individual heavy metal concentrations

Individual elements were examined to detect concentrations of heavy metals above expected background that frequently occur in wetland soils. Mean metal concentrations ($\overline{X}$) for the NWCA sampled population represented by the probability sites, as well as the mean concentrations for the portion of the population above (*X*_↑_) and below (*X*_↓_) the background concentration thresholds, are provided for each measured element, nationally and for the four NWCA Aggregated Ecoregions, in Table 3. Both nationally and for the individual NWCA regions, the percent of the population with heavy metal concentrations below the background concentration thresholds far exceeded that with concentrations above the background concentration thresholds.

Although most heavy metals were not found in concentrations above the background concentration threshold in greater than 5% of the area of the population across the nation, a few elements were found in high concentrations in a relatively high percentage of the estimated area of the sampled population in the West. Copper, antimony, and zinc were considered common in the West and were found in concentrations above the background concentration threshold in 10.3, 39.3, and 5.7% of the sampled wetland area for this region. Furthermore, the mean concentrations above the background concentration threshold of antimony and zinc, especially, in the West tended to be double or more above that of expected background (2.01 ± 0.84 ppm Sb and 406.02 ± 129.76 ppm Zn). However, high mean concentrations for antimony and zinc were found only in a few sites (*n* = 15 and 12, respectively) that represent a large portion of the wetland population, and therefore, these sites largely drive the HMI results in the West (Fig. 7).

Ten of the 12 heavy metals measured were found in less than 5% of the national population in concentrations above the background concentration threshold. However, cadmium and lead were found at concentrations above the established threshold in more than 5% of the wetland area nationally, with cadmium at 6.8% and lead at 11.3% percent of the area, making them the two most common heavy metals nationally. Cadmium was found in the highest concentrations above the background concentration threshold in the Coastal Plains followed by the West with means of 4.35 ± 8.65 and 3.51 ± 0.99 ppm Cd, respectively, but these two regions also had the lowest extent of wetland area with cadmium above the background concentration threshold (0.8% and 4.4% of the wetland area in the Coastal Plains and West, respectively; Supplementary Table 1). While there was far more wetland area in the Eastern Mountains & Upper Midwest and Interior Plains with cadmium above the background concentration threshold (13.3 and 15.9% of the population), the mean cadmium concentrations above the background concentration threshold were much lower than those of the Coastal Plains and West and in fact were only 0.28 and 0.34 ppm Cd above the 1.0-ppm threshold (for Eastern Mountains & Upper Midwest and Interior Plains, respectively).

In addition to cadmium, lead was found in concentrations above the background concentration threshold frequently across the US. The national mean above the background concentration threshold was 57.66 ± 6.70 ppm Pb, although no soils exceeded 400 ppm, the threshold at which US EPA identifies soils as contaminated (i.e., potentially hazardous to human health) (US EPA 2001). Particularly high mean concentrations above the background concentration threshold were found in the Coastal Plains (70.44 ± 17.70 ppm Pb) and West (82.08 ± 53.42 ppm Pb). While the mean above the background concentration threshold for the Coastal Plains represents 3.4% of the area of the Coastal Plains subpopulation (approximately 0.39 ± 0.19 million ha), the mean above the background concentration threshold for the West represents 18.4% of the estimated wetland area in the West subpopulation, or approximately 0.27 ± 0.24 million ha. Despite a lower mean concentration above the background concentration threshold (52.17 ± 6.66 ppm Pb) compared to other regions, the wetland area affected by lead concentrations above the background concentration threshold in the Eastern Mountains & Upper Midwest was large, representing 23.9% or 1.90 ± 0.72 million ha, making lead the most common heavy metal above the background concentrations in wetlands of this region. The frequency of lead in both the Eastern Mountains & Upper Midwest and West, and across the US, prompted us to investigate wetland soil lead concentrations further.

### Soil lead concentrations and possible sources

Given the ranges and means of background soil lead concentrations for the US (e.g., Shacklette and Boerngen 1984; Holmgren et al. 1993), the likelihood of elevated soil lead (i.e., > 35 ppm Pb) originating from natural sources is low. Elevated soil lead concentrations come from two main anthropogenic sources in the US—lead from vehicle emissions and lead-based paint (Mielke and Reagan 1998; Mielke et al. 2007; Alloway 2013). For nearly 70 years, from the 1920s to the mid-1990s, tetraethyl lead was used as an additive to gasoline as an anti-knock agent (Quarles III et al. 1974; Nriagu 1978; see Kovarik 2005 for a discussion on the history of leaded gasoline and human health), and lead was added to paint as a pigment for centuries, with use peaking in the 1920s before it was phased down in the US beginning in 1978 (US CPSC 1977a, b; US HUD 1997; Mielke and Reagan 1998; Mielke et al. 2007). Given the history of lead sources in the US, we hypothesized that wetland soil lead concentrations would be associated with landscape metrics associated with roads and with housing.

**Fig. 8:**
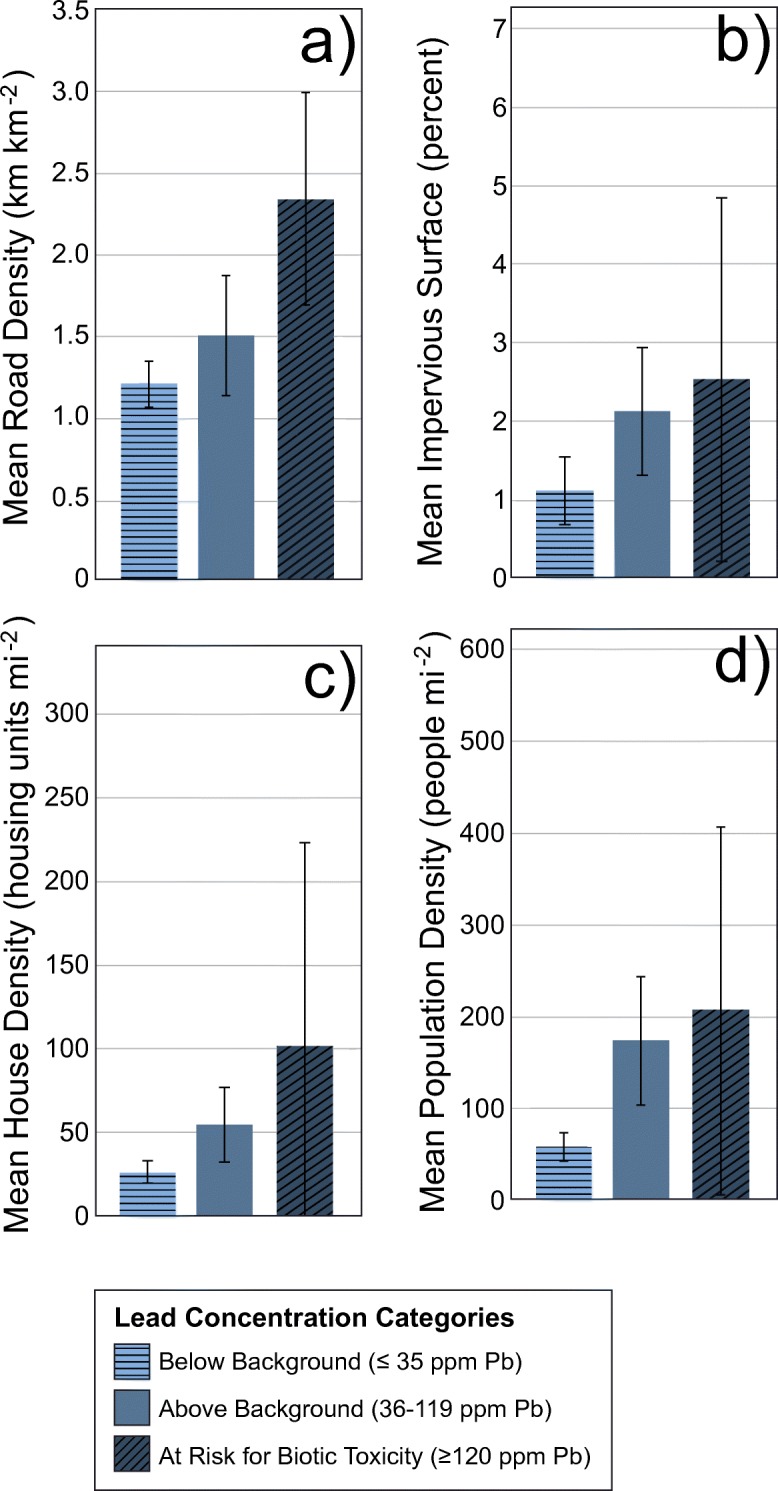
The relationship between lead (Pb) concentration categories and **a** mean road density (km km^−2^), **b** mean impervious surface (%), **c** mean house density (housing units mi^−2^), and **d** mean population density (people mi^−2^) within the 1-km buffer of the wetland point for the national wetland population. The lightest blue, horizontally striped bars represent ≤ 35 ppm Pb; blue, solid bars represent 36–119 ppm Pb; and the darkest blue, diagonally striped bars represent ≥ 120 ppm Pb. Error bars represent 95% CI. The wetland population extent associated with each lead concentration category can be found in Table 4e

Figure 8 shows the relationship between landscape metrics associated with roads and other impervious surfaces and soil lead concentrations (Fig. 8a, b), and the relationship between landscape metrics associated with housing and population and soil lead concentrations (Fig. 8c, d). Nationally, all landscape metrics—road density, impervious surface, housing unit density, and population density—showed a positive trend with the soil lead concentration categories: higher lead concentrations tended to be associated with dense road and housing within the 1-km buffer. However, high variability among sites combined with elevated soil lead concentrations occurring at few numbers of sites (i.e., low sample size with ≥ 120 ppm Pb) made significant relationships among the three lead concentration categories difficult to identify, especially at regional levels.

Mean road density had the strongest relationship with soil lead concentration categories, with a significant difference in road density between soil concentrations ≤ 35 ppm Pb and ≥ 120 ppm Pb. In addition, road densities associated with At Risk for Biotic Toxicity (2.30 ± 0.65 km km^−2^) were nearly twice that of road densities associated with Below Background (1.19 ± 0.14 km km^−2^) (Fig. 8a). The mean road density for the Above Background (36–119 ppm) soil concentrations of Pb was not significantly different from either the Below Background or the At Risk for Biotic Toxicity lead concentration categories. Although soil lead concentrations increased with increasing mean impervious surface, there were no significant differences among the three lead concentration categories (Fig. 8b). Mean housing unit density and mean population density were significantly higher when soil concentrations were 36–119 ppm Pb rather than ≤ 35 ppm Pb, but there was no difference between either lead concentration category and the At Risk for Biotic Toxicity category due to the high variability in housing and population densities associated with soils ≥ 120 ppm Pb (Fig. 8c, d). Mean housing unit density was 53.4% greater and mean population density was 67.1% greater when soil concentrations were 36–119 ppm Pb rather than ≤ 35 ppm Pb.

Regional relationships among landscape metrics and lead concentration categories reveal some interesting trends. Mean road density, impervious surface, housing unit density, and population density (i.e., all landscape metrics) associated with soil concentrations ≤ 35 ppm Pb (Below Background) tended to be highest in the West compared to the other three regions, although there is considerable variation associated with the means (Tables 4a-d). This suggests that sites in the West especially can have soil lead concentrations below background but high amounts of road density, impervious surface, house density, and population density within the 1-km buffer, perhaps reflecting later anthropogenic development than east of the Rocky Mountain range. The Coastal Plains and Interior Plains tended to have the highest means for all landscape metrics associated with soil concentrations 36–119 ppm Pb (Above Background). And for all landscape metrics measured in the Eastern Mountains & Upper Midwest, there was no difference in means between Below Background and Above Background lead concentration categories, but there was a notable and significant increase in the means at the At Risk for Biotic Toxicity lead concentration category. However, there were only five sites representing 0.01 million ha of wetland area (or 0.1% of the Eastern Mountains & Upper Midwest population) that had soil concentrations ≥ 120 ppm Pb. In fact, high concentrations of soil lead (i.e., ≥ 120 ppm Pb) were only found in 20 sites nationally (representing an areal extent of 0.11 million ha, or about 0.4% of the wetland population) (Table 4e).

**Table 4:** Population means ± 95% confidence interval (CI) for a) road density (km km^-2^), b) impervious surface (percent), c) house density (housing units mi^-2^), and d) population density (people mi^-2^) within the 1-km buffer of the wetland point and the associated lead (Pb) concentration categories in four NWCA Aggregated Ecoregions (see Figure 2 for abbreviations). e) Extent (10^6^ ha) of the wetland population represented by each lead concentration category (for the nation and each region), including the 95% CI, the percent of the population represented, and the number (n) of probability sites sampled. NA signifies that there were no sites in the corresponding lead concentration category

NWCA Aggregated Ecoregion	Lead Concentration Category		
	Below Background (≤ 35 ppm Pb)	Above Background (36-119 ppm Pb)	At Risk for Biotic Toxicity (≥ 120 ppm Pb)
a) Road Density (km km^-2^)			
National	1.19 ± 0.14	1.48 ± 0.37	2.30 ± 0.65
CPL	1.19 ± 0.15	2.43 ± 1.50	1.53 ± 0.46
EMU	1.16 ± 0.39	1.37 ± 0.40	7.40± 1.36
IPL	1.08 ± 0.13	1.85 ± 1.03	NA
W	1.51 ± 0.57	0.84 ± 0.54	2.48 ± 0.673
b) Impervious Surface (percent)			
National	1.11 ± 0.43	2.12 ± 0.82	2.54 ± 2.31
CPL	0.90 ± 0.40	3.87 ± 3.23	1.30 ± 1.05
EMU	1.14 ± 0.80	1.94 ± 0.96	21.22 ± 10.31
IPL	0.63 ± 0.31	3.09 ± 3.75	NA
W	3.80 ± 4.78	0.69 ± 0.68	0.46 ± 0.22
c) House Density (housing units mi^-2^)			
National	25.49 ± 6.77	54.69 ± 21.91	101.52 ± 121.50
CPL	20.99 ± 5.03	194.73 ± 162.56	32.44 ± 32.58
EMU	32.16 ± 18.94	30.39 ± 14.42	1061.14 ± 621.61
IPL	21.21 ± 13.02	107.98 ± 120.41	NA
W	42.28 ± 50.13	32.75 ± 51.69	3.89 ± 0.46
d) Population Density (people mi^-2^)			
National	57.08 ± 15.26	174.22 ± 69.84	205.81 ± 200.88
CPL	44.11 ± 7.43	478.60 ± 407.79	84.39 ± 63.91
EMU	75.46 ± 45.29	121.41 ± 67.98	2009.15 ± 851.66
IPL	48.95 ± 29.01	343.51 ± 424.49	NA
W	101.94 ± 117.26	104.37 ± 157.40	7.85 ± 2.13
e) Wetland Population Extent (absolute area 10^6^ ha (percent of population), number of sites)			
National	20.82 ± 2.03 (82.7%), n=724	2.55 ± 0.79 (10.1%), n=130	0.11 ± 0.07 (0.4%), n=20
CPL	11.27 ± 1.38 (90.1%), n=407	0.33 ± 0.19 (2.6%), n=50	0.06 ± 0.05 (0.5%), n=12
EMU	6.06 ± 1.24 (75.0%), n=93	1.90 ± 0.73 (23.5%), n=53	0.01 ± 0.01 (0.1%), n=5
IPL	2.29 ± 0.70 (73.8%), n=104	0.10 ± 0.07 (3.1%), n=7	NA
W	1.20 ± 0.37 (80.9%), n=120	0.23 ± 0.23 (15.7%), n=20	0.04 ± 0.04 (2.5%), n=3

#### Lead and vehicles

In this study, we found positive relationships between soil lead concentration and road density, supporting our hypothesis that higher soil lead concentrations are associated with increased intensity of impacts related to roads in the 1-km buffer. Roads have many negative impacts on ecosystems, including alteration of the chemical environment (Trombulak and Frissell 2000), and vehicles are one of the major sources of modern-day lead contamination in soils (Alloway 2013). Concentrations in dust collected directly from streets in the mid-1970s were as high as 52,000 ppm Pb (Harrison 1979), although roadside soil lead concentrations tend to be orders of magnitude less. Elevated lead concentrations in roadside soils vary depending on distance from the road, traffic intensity, and prevailing wind direction and have been reported in concentrations ranging from just a few ppm above background to more than 30 times background (Page and Ganje 1970; Page et al. 1971; Smith 1976; Tong 1990). It has been shown that soil lead concentrations in large cities can be 10 to 100 times greater than that of smaller cities and towns due to traffic volumes (Mielke and Reagan 1998; Mielke 1999). Lead particulates from both exhaustive and non-exhaustive sources contribute to roadside contamination, with primary vehicular-based sources as (1) historical (i.e., pre-1974) emissions from leaded gasoline, which contained 2–4 g of tetraethyl lead per gallon of gasoline (Quarles III et al. 1974; Nriagu 1978), and (2) dust from brake linings, which have been shown to be comprised of up to 12 wt.% Pb (Thorpe and Harrison 2008; Grigoratos and Martini 2015). Lead contamination of plants and soils adjacent to highways has been studied thoroughly (e.g., Cannon and Bowles 1962; Page et al. 1971; Goldsmith et al. 1976), and elevated soil lead concentrations have been reported even as far as 1 km from major highways with high vehicle densities (Page and Ganje 1970), presumably due to transport via airborne ultrafine particles (Alloway 2013).

#### Lead and paint

Our data show positive relationships between soil lead concentration and housing unit density and population density, supporting our hypothesis that higher soil lead concentrations are associated with increased intensity of impacts related to housing in the 1-km buffer. It is estimated that equal amounts of lead have been introduced to the environment in the US from lead-based paint, widely used into the late 1970s, and from leaded gasoline, roughly equally 6 million raw tonnes (Mielke 1999; Mielke et al. 2007; Alloway 2013). Lead paint can be introduced into the soil from deterioration or by removal (e.g., sanding, stripping) (Mielke et al. 1997), although the highest soil lead concentrations occur near drip lines and foundations of wood-sided buildings (Schmitt et al. 1988; Rogers et al. 1993). Fine dust particulates (i.e., pulverized particles) from lead-based paint, on the other hand, have a propensity to be aerially redistributed, which can impact soil lead concentrations at a distance from housing (Mielke 1999). While some studies suggest that lead from urban housing is as important a source of soil lead contamination as leaded gasoline (Bertinuson and Clark 1973), other studies have shown that soil lead concentrations in inner cities—even with brick housing—tend to be orders of magnitude greater than rural areas that have old housing with lead-based paint (Mielke et al. 1997; Mielke 1999). These latter studies suggest that even though paint can be a significant source of soil lead, vehicular emissions are the main contributor of soil lead. Housing unit density and population density are not unrelated to road density and impervious surfaces, as infrastructure (i.e., roads and housing) increases with the number of people in an area. Therefore, the relationship that we see in our study with soil lead concentration and housing unit density and population density may reflect co-occurring road density and impervious surfaces, at least in part.

### Lead sorption as an ecosystem service

After decades of discharging lead into our environment, the US has enforced several standards and regulations to reduce the use of lead in vehicular and household materials. In 1974, the US government began enacting a series of federal standards to phase down lead additives to gasoline, and on January 1, 1996, leaded gasoline was banned for on-road vehicles under the authority of the Clean Air Act (Newell and Rogers 2003). In 1978, the use of lead paint for residential houses was banned in the US by the Consumer Product Safety Commission (US CPSC 1977a, b; US HUD 1997). Most recently, the US EPA released a Memorandum of Understanding in partnership with several major automotive part companies to voluntarily reduce the use of lead and its components (among other metals, such as copper) in brake pads by 2025 under the authorities of the Clean Water Act and the National Environmental Policy Act (US EPA 2015b).

Given these regulations and the resulting decrease of lead being introduced into the environment, it would be reasonable to expect low lead concentrations in the surface horizons of soil in this study, which typically represent the youngest horizons. However, our data show that the uppermost soil horizons can have large accumulations of lead, despite efforts to curb lead use. It is possible that the uppermost soils horizons are reflecting legacy lead deposition given the correlation with roads and housing, perhaps from leaded gasoline and lead-based paint. Even though the wetland soils sampled for this study represented the surface horizons (typically beginning within 2 cm from the surface), the sample also represented a composite from the entire surface horizon, which had a mean thickness of approximately 30 cm across the nation. Depending on vertical accretion rates at an individual site, the uppermost horizon may represent a time period of months or decades—perhaps even centuries—of soil development. Vertical accretion rates vary widely across wetlands in the US, with studies reporting Gulf of Mexico coastal wetlands gaining 0.18 to 0.89 cm yr^−1^ (Calloway et al. 1997), 0.30 to 1.3 cm yr^−1^ for salt marshes and natural and canal waterways in Louisiana (Cahoon and Turner 1989), 0.36 cm yr^−1^ on average for salt marshes in the Pacific Northwest (Thom 1992), between 0.9 and 1.4 cm yr^−1^ for newly created (i.e., < 15 years old) riverine wetlands (Anderson and Mitsch 2006; Bernal and Mitsch 2013), 0.06 to 0.22 cm yr^−1^ for depressional wetlands, and 0.03 to 0.19 mm yr^−1^ for floodplain forested wetlands (Craft and Casey 2000). Given that soils typically build slowly in wetlands, both historical and contemporary sources of lead (and other heavy metals) are likely to be represented in our data.

Lead is relatively immobile in the soil compared to other heavy metals (US EPA 2005; Alloway 2013), with estimated residence times of anthropogenic lead at the catchment scale on the order of centuries to millennia (Tipping et al. 2006). High concentrations of soil organic matter, well-buffered soils, and the reduced conditions typical of many wetlands make them optimal sites for lead sorption. Lead mobility is largely dictated by the pH and organic matter content of soils, as the humic fraction can strongly adsorb lead at a pH of 4 and above (Bunzl et al. 1976; Kerndorff and Schnitzer 1980; Alloway 2013). Lead can also be strongly bound to clay minerals and iron oxides in the absence of substantial organic matter (Hildebrand and Blum 1974a, b; Scrudato and Estes 1975; Alloway 2013).

Soil mobility of lead is affected by many competing factors and conditions. While pH is the dominating factor for determining mobility of heavy metals in soils, the redox condition can also influence heavy metal mobility from sediments. Laboratory studies have shown that lead mobility especially is affected during reducing conditions due to transformations of available carbon and the dissolution of manganic and ferric oxides (Grybos et al. 1996; Charlatchka and Cambier 2000). In contrast, several studies have shown that lead is more bioavailable under oxidized conditions (Gambrell 1994) or suggest that solid compounds of bound heavy metals (particularly by sulfidic compounds) tend to be stable under sustained reduced conditions, especially at pH of 7 and above (Calmano et al. 1993; Hawkins et al. 1997). Although organically complexed lead can be solubilized and transported on dissolved organic carbon (Bergkvist 2001), soils represent the most concentrated physical pool of metals in aquatic environments (Luoma 1983). Several field-based studies have found that wetland soils can act as traps for mobilized lead. For example, Turner et al. (1985) conducted an investigation of lead concentrations, fluxes, and storage in the New Jersey Pine Barrens and found that lowland muck soils and vegetation sequestered 98% of lead incoming to the watershed, with very little being exported to streams. In a 7-year study to investigate the potential of natural salt marshes to act as waste treatment systems, Giblin et al. (1980) introduced heavy metal-laden sewage sludge and found that high marsh areas retained up to 100% of introduced lead in a form unavailable (i.e., immobilized by sediments) to plants or animals. Beining and Otte (1997) reported that for a natural, organic-rich wetland receiving mine effluent for over a century in Ireland, lead concentrations in pore water dramatically decreased with distance from the source, presumably due to sorption to wetland soil organic matter, which had concentrations ranging from 400 to 17,600 ppm Pb.

There is a wide diversity of hydrology and biogeochemistry in wetlands across the US, and obviously, not all individual wetlands are well suited for trapping lead. While this study was not designed to specifically address wetland soil biogeochemistry and soil heavy metal mobility, some of the data we collected suggests that it may be likely that a large portion of wetlands in the US are providing a critical ecosystem service by trapping lead. Table 5 presents the mean percent soil organic carbon (SOC) and the mean pH, along with the mean soil lead concentration for the nation and each of the four NWCA Aggregated Ecoregions. The average pH is 4.38 ± 0.10 for wetlands in the US, with increasing pH from east to west, and soil organic carbon ranging from 5.33 to 33.50% SOC depending on the region. Mean soil concentration was 15.82 ± 1.46 ppm Pb in the Interior Plains, where there was also very little soil organic carbon (6.39 ± 1.43% SOC) compared to the Eastern Mountains & Upper Midwest and West (23.83 ± 4.08 and 24.81 ± 11.43 ppm Pb, respectively). While Eastern Mountains & Upper Midwest had the highest soil organic carbon content (33.50 ± 4.32% SOC), largely due to the number of peatlands found in the Eastern Mountains & Upper Midwest driving the exceptionally high soil organic carbon mean for this region (Nahlik and Fennessy 2016), the West had the lowest organic carbon content (5.33 ± 1.46% SOC). Given the high densities of roads and housing in the Eastern Mountains & Upper Midwest, which, in this study, are associated with elevated soil lead concentrations, it is fortuitous that, on average, wetlands in the Eastern Mountains & Upper Midwest have high soil organic carbon content that can support lead immobilization. It is possible that lead mobilization might be a larger issue in the West, where there is not as much soil organic carbon to help bind lead (although soils tend to have higher pH in the West than the Eastern Mountains & Upper Midwest, which promotes lead immobilization). Studies have shown that even in sediments at a neutral pH or above, constant oxidation (through mixing), can lower soil pH to under 3, resulting in mobilized heavy metals (Gambrell 1994); thus, maintaining our wetlands in good condition without disturbing the soils is critical to preventing mobilization of any bound lead or other heavy metals.

**Table 5:** Mean soil pH, soil organic carbon (%), and soil lead (ppm Pb), and areal extent (10^6^ ha) for the national wetland population and the four NWCA Aggregated Ecoregions. 95% CI is reported with each mean. n-values (i.e., number of probability sites) on which areal extent is based are 874, 469, 151, 111, and 143, for the conterminous US, CPL, EMU, IPL and W, respectively

NWCA Aggregated Ecoregion	Soil pH	Soil Organic Carbon (%)	Soil Lead(ppm Pb)	Extent(10^6^ ha)
Conterminous US	4.38 ± 0.10	17.17 ± 1.88	20.15 ± 1.73	23.48 ± 1.90
Coastal Plains (CPL)	4.26 ± 0.12	9.72 ± 1.71	17.95 ± 1.35	11.67 ± 1.35
Eastern Mts & Upper Midwest (EMU)	4.39 ± 0.18	33.50 ± 4.32	23.83 ± 4.08	7.96 ± 1.08
Interior Plains (IPL)	5.44 ± 0.47	6.39 ± 1.43	15.82 ± 1.46	2.38 ± 0.69
West (W)	5.75 ± 0.23	5.33 ± 1.46	24.81 ± 11.43	1.46 ± 0.34

## Summary and conclusions

The 2011 NWCA was the first probability-based survey of the condition of our nation’s wetlands using data gathered in the field. Using a single, national background concentration threshold for each of 12 measured elements in wetland soils, we investigated human-mediated heavy metal additions to our wetland ecosystems using two approaches: (1) an aggregated index, the HMI, that indicates the magnitude of human activities that could negatively affect the wetland population over national and regional scales and (2) individual concentrations of elements that aid in our understanding of detailed patterns of heavy metal concentrations in soils and in identifying heavy metals in aquatic soils that occur frequently in concentrations above expected background. The HMI results suggest that the majority of assessed wetland area in the nation had low heavy metal loads in surface soil horizons, thus, indicating that the sampled wetland population was not strongly affected by recent human activities that result in heavy metal deposition/accumulation. The evaluation of individual heavy metal concentrations in wetlands showed that the West had several common heavy metals, including copper, antimony, and zinc, while at the national scale, two heavy metals were common—cadmium and lead. Even though lead was most common in wetland soils in this study compared to other heavy metals, soil concentrations were typically low (< 35 ppm Pb), with only 0.4% of the estimated area in the sampled population with concentrations exceeding 120 ppm Pb, a plant toxicity threshold. There were no measured soil concentrations exceeding 400 ppb Pb—the threshold for indicating a potential hazard to human health (US EPA 2001). Our data showed a positive relationship between lead concentration categories and road density metrics and housing unit metrics, suggesting that vehicular deposition and, to a lesser degree lead-based paints, may be sources of lead into wetland soils. We found that mean lead concentrations above expected natural background (i.e., > 35 ppm Pb) were highest in the Eastern Mountains & Upper Midwest, with a mean elevated soil concentration of 52.17 ± 6.66 ppm Pb (Table 3). Of the four NWCA Aggregated Ecoregions, the Eastern Mountains & Upper Midwest also had the highest mean road density, impervious surface, house density, and population density associated with soil concentrations ≥ 120 ppm Pb. These results suggest that wetlands in the Eastern Mountains & Upper Midwest may be impacted by the numerous large cities, which are associated with high road densities and perhaps older homes that are more likely to have lead-based paint that could be pulverized and distributed. The Eastern Mountains & Upper Midwest also has the highest soil organic carbon content in wetlands (33.50 ± 4.32%; Table 5), which could be immobilizing lead. In the Eastern Mountains & Upper Midwest and across the nation, it is critical to maintain our wetlands in good condition so that any immobilized lead remains unavailable and so the wetlands may continue to act as potential filters for soil heavy metals, and lead in particular.

## Electronic supplementary material

ESM 1 (DOCX 765 kb)
