# Supplementary material for: Use of national-scale data to examine human-mediated additions of heavy metals to wetland soils of the US
Source: Environ Monit Assess. 2019 Jun 20;191(Suppl 1):336. doi: 10.1007/s10661-019-7315-5 (PMC6586720; doi:10.1007/s10661-019-7315-5)
Supplement: Supplementary file 1 — (DOCX 765 kb) [file 10661_2019_7315_MOESM1_ESM.docx]

**Supplementary Table 1.** Extent (10^6^ ha) of the wetland population associated with means of 12 elements measured in the uppermost soil horizon (see Table 3). $X_{\downarrow}$ and $X_{\uparrow}$ represent the extent for sites with heavy metal concentrations above and below the background concentration threshold, respectively. Total extent and number of probability sties for $\bar{X}$ can be found in Table 5. The full names of the elements are reported in the Methods and in Table 2. 95% CI is reported with the extent, and the number of probability sites associated with the extent is reported in parentheses. NA signifies that there were no sites with heavy metal concentrations above the background concentration threshold.

| **Element** | 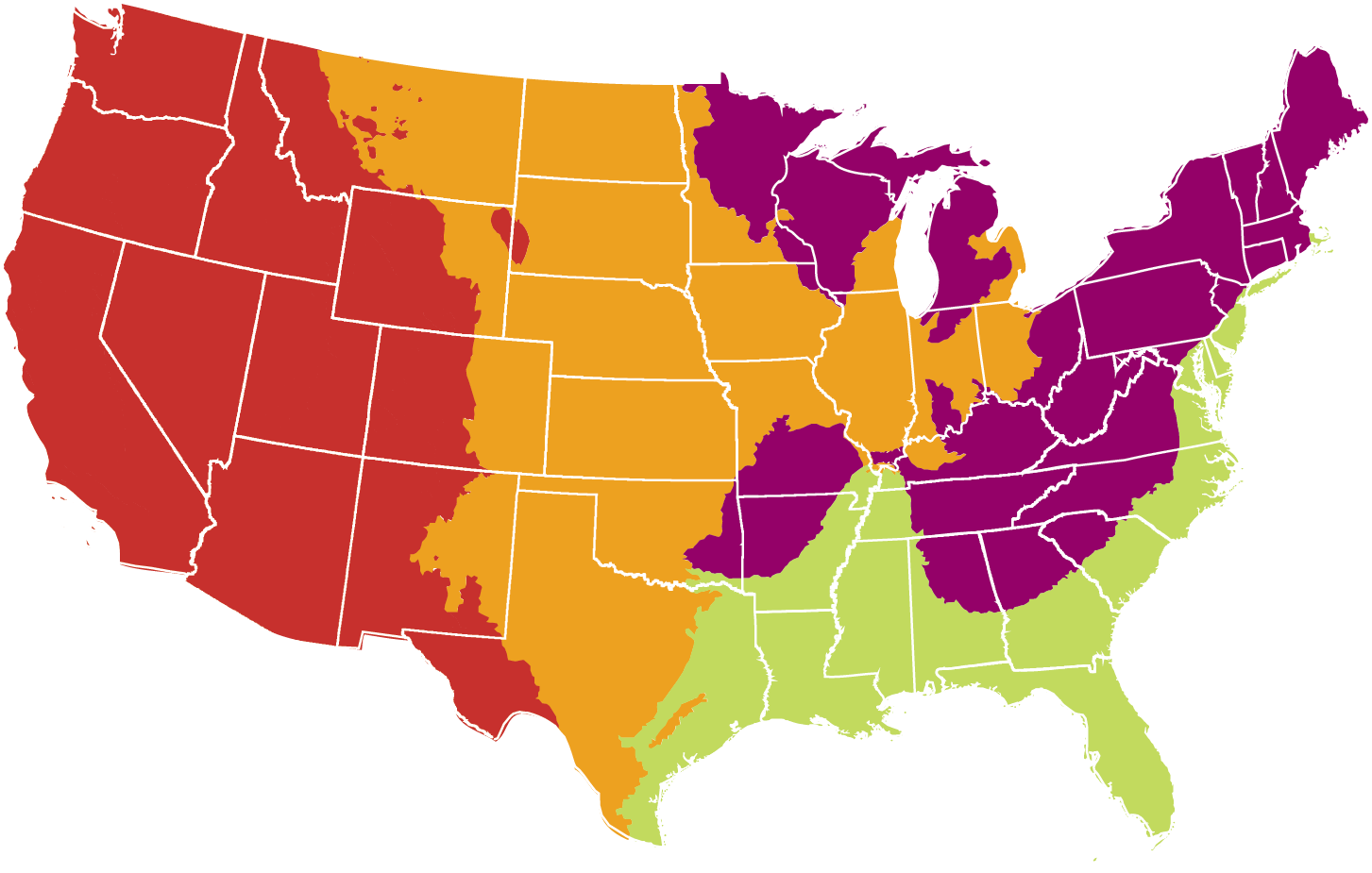  **Conterminous US** | 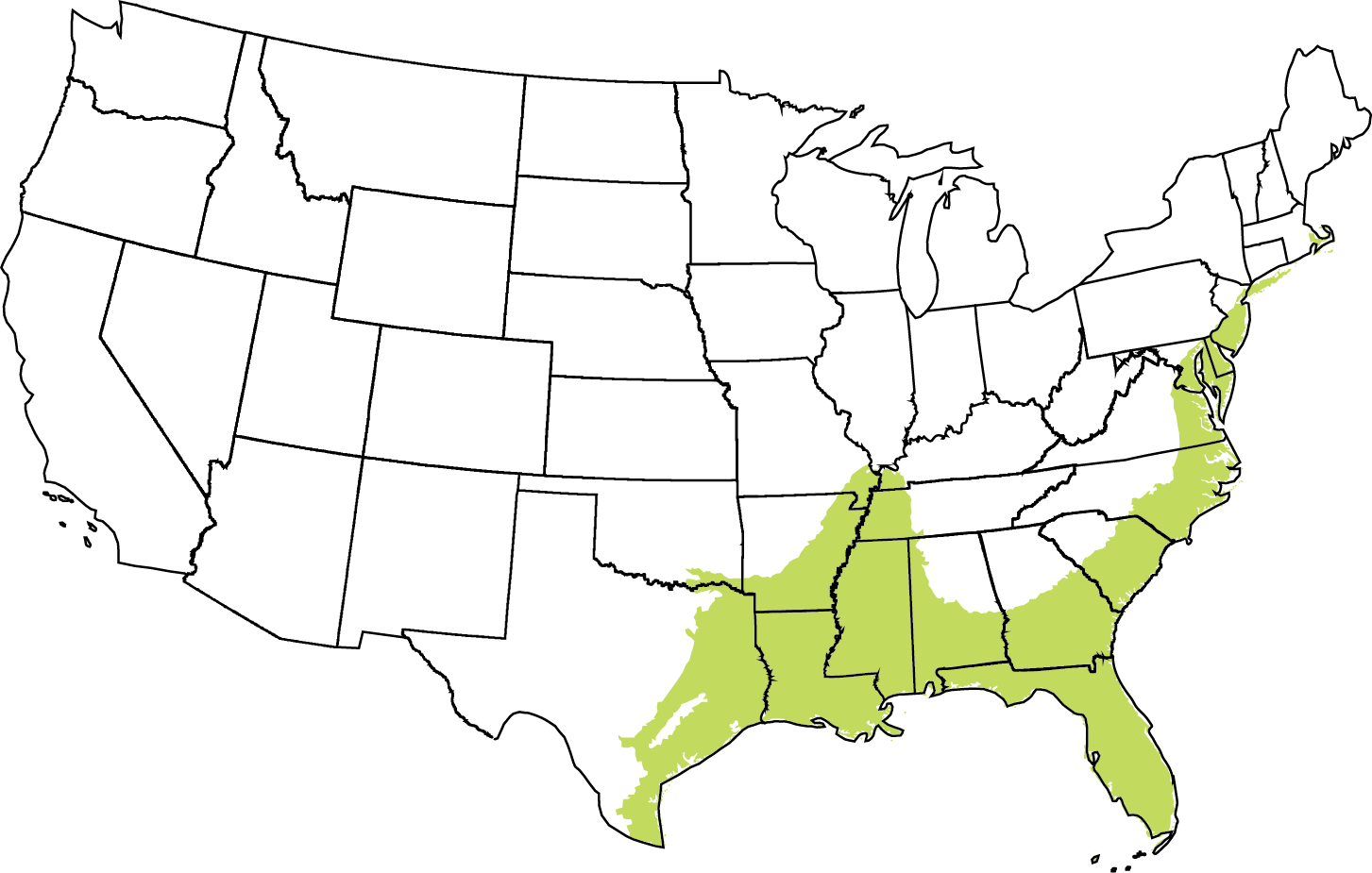  **Coastal Plains (CPL)** | 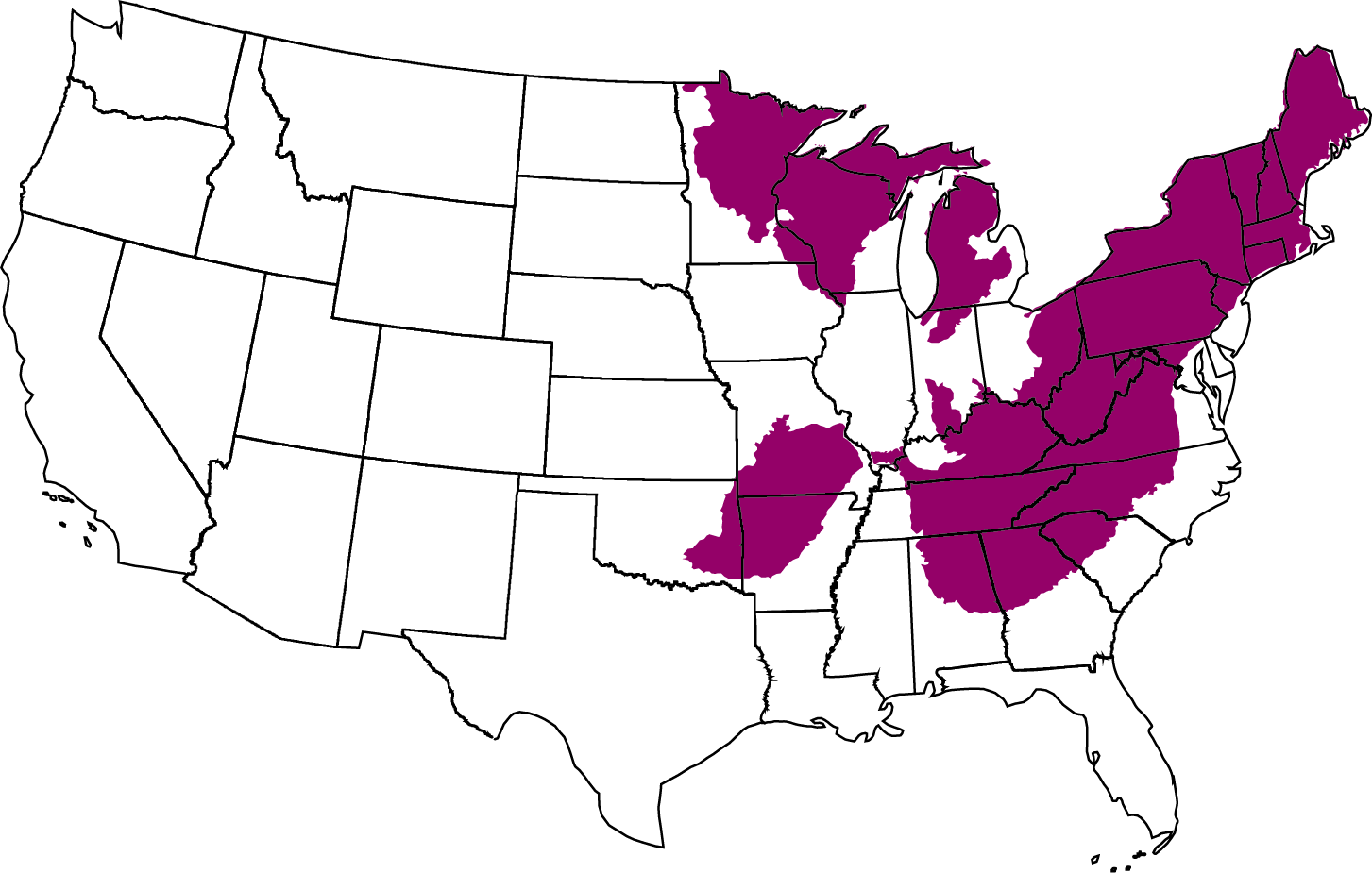  **Eastern Mts & Upper Midwest (EMU)** | 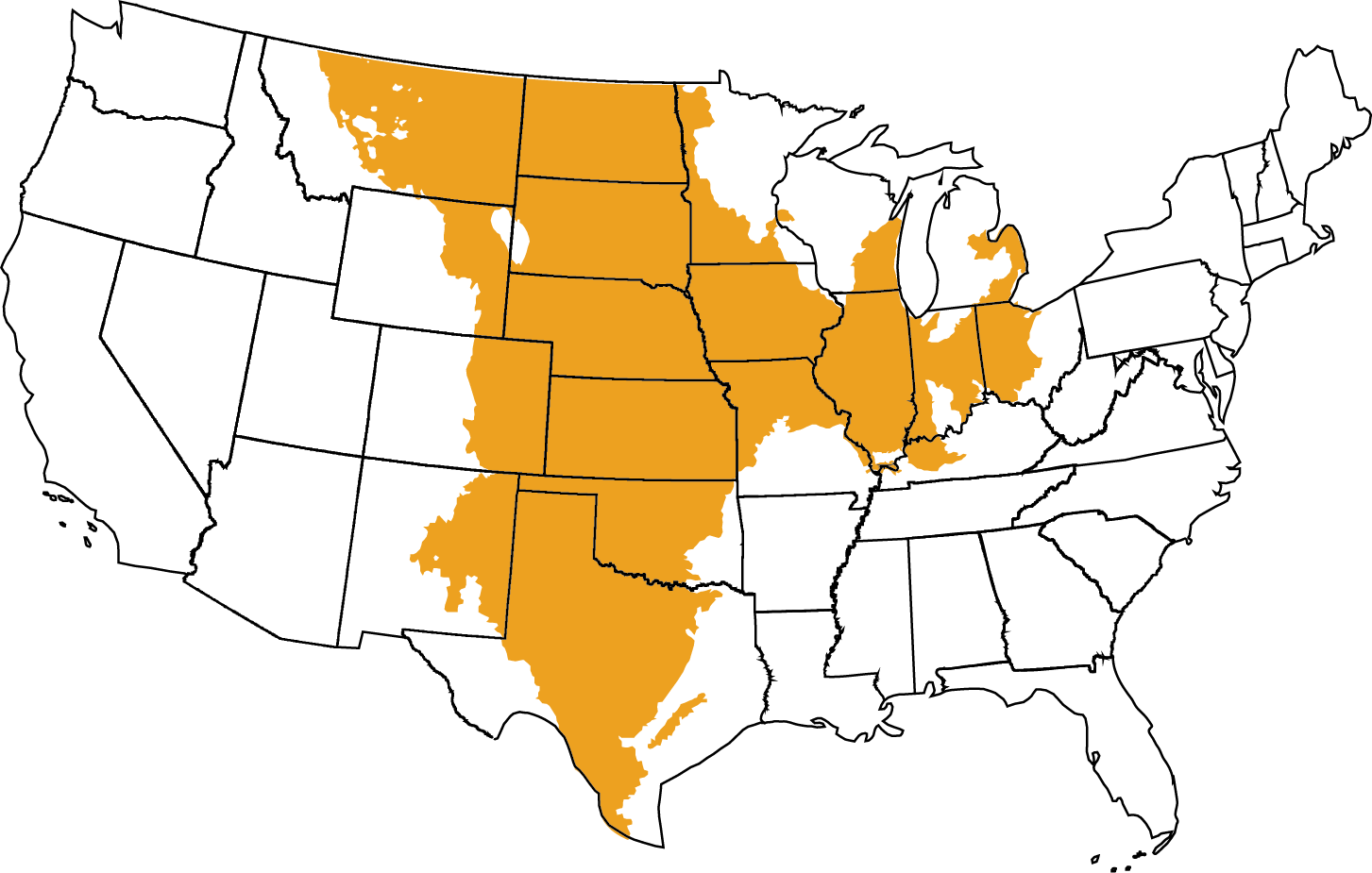  **Interior Plains (IPL)** | 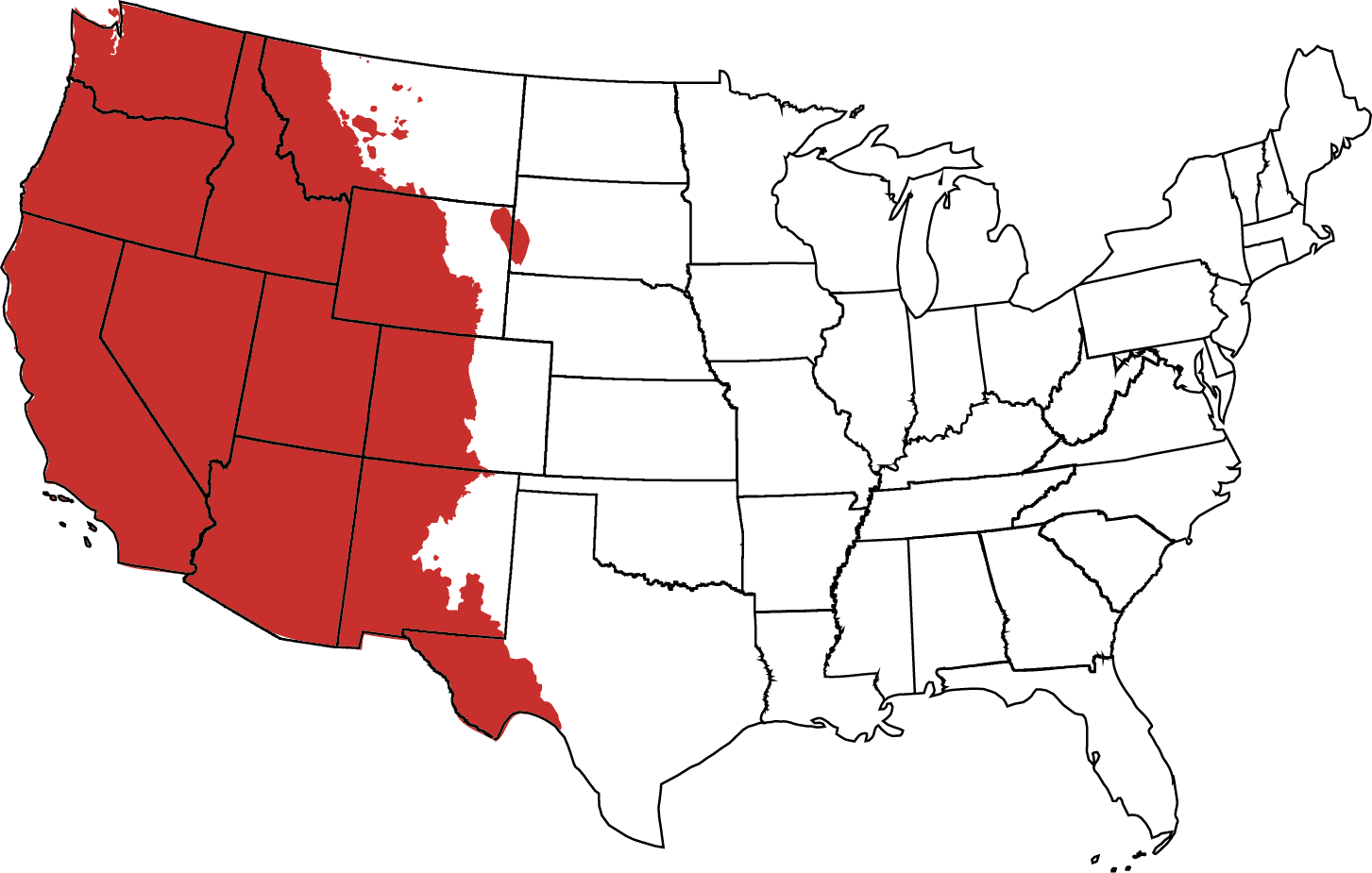  **West (W)** |
| --- | --- | --- | --- | --- | --- |
| **Ag** | $X_{\downarrow}$ 23.43 ± 1.90 (867)  $X_{\uparrow}$ 0.04 ± 0.05 (7) | $X_{\downarrow}$ 11.66 ± 1.35 (466)  $X_{\uparrow}$ 0.01 ± 0.01 (3) | $X_{\downarrow}$ 7.96 ± 1.08 (149)  $X_{\uparrow}$ 0.00 ± 0.00 (2) | $X_{\downarrow}$ 2.38 ± 0.69 (111)  $X_{\uparrow}$ NA | $X_{\downarrow}$ 1.43 ± 0.34 (141)  $X_{\uparrow}$ 0.03 ± 0.04 (2) |
| **Cd** | $X_{\downarrow}$ 21.88 ± 1.90 (836)  $X_{\uparrow}$ 1.60 ± 0.88 (38) | $X_{\downarrow}$ 11.58 ± 1.35 (467)  $X_{\uparrow}$ 0.09 ± 0.11 (2) | $X_{\downarrow}$ 6.90 ± 1.10 (129)  $X_{\uparrow}$ 1.06 ± 0.73 (22) | $X_{\downarrow}$ 2.00 ± 0.64 (106)  $X_{\uparrow}$ 0.38 ± 0.44 (5) | $X_{\downarrow}$ 1.40 ± 0.34 (134)  $X_{\uparrow}$ 0.06 ± 0.05 (9) |
| **Co** | $X_{\downarrow}$ 23.17 ± 1.92 (863)  $X_{\uparrow}$ 0.31 ± 0.35 (11) | $X_{\downarrow}$ 11.67 ± 1.35 (468)  $X_{\uparrow}$ 0.00 ± 0.00 (1) | $X_{\downarrow}$ 7.67 ± 1.10 (146)  $X_{\uparrow}$ 0.30 ± 0.34 (5) | $X_{\downarrow}$ 2.38 ± 0.69 (111)  $X_{\uparrow}$ NA | $X_{\downarrow}$ 1.46 ± 0.34 (138)  $X_{\uparrow}$ 0.01 ± 0.01 (5) |
| **Cr** | $X_{\downarrow}$ 23.45 ± 1.91 (870)  $X_{\uparrow}$ 0.03 ± 0.03 (4) | $X_{\downarrow}$ 11.64 ± 1.36 (466)  $X_{\uparrow}$ 0.03 ± 0.06 (3) | $X_{\downarrow}$ 7.96 ± 1.08 (150)  $X_{\uparrow}$ 0.00 ± 0.00 (1) | $X_{\downarrow}$ 2.38 ± 0.69 (111)  $X_{\uparrow}$ NA | $X_{\downarrow}$ 1.46 ± 0.34 (143)  $X_{\uparrow}$ NA |
| **Cu** | $X_{\downarrow}$ 22.93 ± 1.92 (821)  $X_{\uparrow}$ 0.55 ± 0.41 (53) | $X_{\downarrow}$ 11.54 ± 1.36 (461)  $X_{\uparrow}$ 0.13 ± 0.13 (8) | $X_{\downarrow}$ 7.71 ± 1.10 (145)  $X_{\uparrow}$ 0.26 ± 0.32 (6) | $X_{\downarrow}$ 2.37 ± 0.69 (110)  $X_{\uparrow}$ 0.01 ± 0.02 (1) | $X_{\downarrow}$ 1.31 ± 0.34 (105)  $X_{\uparrow}$ 0.15 ± 0.03 (38) |
| **Ni** | $X_{\downarrow}$ 23.48 ± 1.90 (874)  $X_{\uparrow}$ NA | $X_{\downarrow}$ 11.67 ± 1.35 (469)  $X_{\uparrow}$ NA | $X_{\downarrow}$ 7.96 ± 1.08 (151)  $X_{\uparrow}$ NA | $X_{\downarrow}$ 2.38 ± 0.69 (111)  $X_{\uparrow}$ NA | $X_{\downarrow}$ 1.46 ± 0.34 (143)  $X_{\uparrow}$ NA |
| **Pb** | $X_{\downarrow}$ 20.82 ± 2.04 (724)  $X_{\uparrow}$ 2.26 ± 0.79 (150) | $X_{\downarrow}$ 11.27 ± 1.37 (407)  $X_{\uparrow}$ 0.39 ± 0.19 (62) | $X_{\downarrow}$ 6.06 ± 1.24 (93)  $X_{\uparrow}$ 1.90 ± 0.72 (58) | $X_{\downarrow}$ 2.29 ± 0.69 (104)  $X_{\uparrow}$ 0.10 ± 0.07 (7) | $X_{\downarrow}$ 1.20 ± 0.37 (120)  $X_{\uparrow}$ 0.27 ± 0.24 (23) |
| **Sb** | $X_{\downarrow}$ 22.60 ± 1.89 (839)  $X_{\uparrow}$ 0.87 ± 0.31 (35) | $X_{\downarrow}$ 11.48 ± 1.35 (455)  $X_{\uparrow}$ 0.18 ± 0.14 (14) | $X_{\downarrow}$ 7.85 ± 1.07 (145)  $X_{\uparrow}$ 0.12 ± 0.15 (6) | $X_{\downarrow}$ 2.38 ± 0.69 (111)  $X_{\uparrow}$ NA | $X_{\downarrow}$ 0.89 ± 0.27 (128)  $X_{\uparrow}$ 0.58 ± 0.22 (15) |
| **Sn** | $X_{\downarrow}$ 23.45 ± 1.90 (871)  $X_{\uparrow}$ 0.03 ± 0.04 (3) | $X_{\downarrow}$ 11.64 ± 1.35 (467)  $X_{\uparrow}$ 0.03 ± 0.04 (2) | $X_{\downarrow}$ 7.96 ± 1.08 (151)  $X_{\uparrow}$ NA | $X_{\downarrow}$ 2.38 ± 0.69 (111)  $X_{\uparrow}$ NA | $X_{\downarrow}$ 1.46 ± 0.34 (142)  $X_{\uparrow}$ 0.00 ± 0.00 (1) |
| **V** | $X_{\downarrow}$ 23.48 ± 1.90 (872)  $X_{\uparrow}$ 0.00 ± 0.00 (2) | $X_{\downarrow}$ 11.67 ± 1.35 (469)  $X_{\uparrow}$ NA | $X_{\downarrow}$ 7.96 ± 1.08 (151)  $X_{\uparrow}$ NA | $X_{\downarrow}$ 2.38 ± 0.69 (111)  $X_{\uparrow}$ NA | $X_{\downarrow}$ 1.46 ± 0.34 (141)  $X_{\uparrow}$ 0.00 ± 0.00 (2) |
| **W** | $X_{\downarrow}$ 23.31 ± 1.90 (858)  $X_{\uparrow}$ 0.17 ± 0.12 (16) | $X_{\downarrow}$ 11.53 ± 1.35 (461)  $X_{\uparrow}$ 0.14 ± 0.11 (8) | $X_{\downarrow}$ 7.94 ± 1.08 (144)  $X_{\uparrow}$ 0.02 ± 0.02 (7) | $X_{\downarrow}$ 2.38 ± 0.69 (111)  $X_{\uparrow}$ NA | $X_{\downarrow}$ 1.46 ± 0.34 (142)  $X_{\uparrow}$ 0.01 ± 0.01 (1) |
| **Zn** | $X_{\downarrow}$ 22.53 ± 1.93 (812)  $X_{\uparrow}$ 0.95 ± 0.44 (62) | $X_{\downarrow}$ 11.22 ± 1.35 (442)  $X_{\uparrow}$ 0.45 ± 0.29 (27) | $X_{\downarrow}$ 7.61 ± 1.13 (136)  $X_{\uparrow}$ 0.35 ± 0.33 (15) | $X_{\downarrow}$ 2.32 ± 0.70 (103)  $X_{\uparrow}$ 0.06 ± 0.04 (8) | $X_{\downarrow}$ 1.38 ± 0.34 (131)  $X_{\uparrow}$ 0.08 ± 0.06 (12) |
